# Process Chain Development for the Fabrication of Three-Dimensional Braided Oxide Ceramic Matrix Composites

**DOI:** 10.3390/ma14216338

**Published:** 2021-10-23

**Authors:** Martin Kolloch, Georg Puchas, Niels Grigat, Ben Vollbrecht, Walter Krenkel, Thomas Gries

**Affiliations:** 1 Institut für Textiltechnik (ITA), RWTH Aachen University, 52074 Aachen, Germany; Niels.Grigat@ita.rwth-aachen.de (N.G.); Ben.Vollbrecht@ita.rwth-aachen.de (B.V.); thomas.gries@ita.rwth-aachen.de (T.G.); 2 Chair of Ceramic Materials Engineering (CME), University of Bayreuth, 95447 Bayreuth, Germany; Georg.Puchas@uni-bayreuth.de (G.P.); walter.krenkel@uni-bayreuth.de (W.K.)

**Keywords:** oxide fibers, bobbin development, 3D braiding, ceramic matrix composites

## Abstract

Fiber composites with a three-dimensional braided reinforcement architecture have higher fiber volume content and Z-fiber content compared to a two-dimensional braided reinforcement architecture; as a result, the shear strength increases. Porous oxide fiber composites (OFCs) have the inherent weakness of a low interlaminar shear strength, which can be specifically increased by the use of a three-dimensional fiber reinforcement. In this work, the braiding process chain for processing highly brittle oxide ceramic fibers is modified; as a consequence, a bobbin, which protects the filament, is developed and quantitatively evaluated on a test rig with regard to tension and filament breakage. Subsequently, a braiding process is designed which takes into account fiber-protecting aspects, and a three-dimensional reinforced demonstrator is produced and tested. After impregnation with an Al_2_O_3_-ZrO_2_ slurry, by either a prepreg process or a vacuum-assisted process, as well as subsequent sintering, the three-dimensional braid-reinforced OFC exhibits an interlaminar shear strength (ILSS) which is higher than that of two-dimensional braid- or fabric-reinforced samples by 64–95%. The influence of the manufacturing process on the relative macropore content is investigated and correlated with the mechanical properties.

## 1. Introduction

The Climate Protection Plan 2050 for Germany envisages a reduction in greenhouse gases by at least 40% by 2050 compared to the levels in 1990 [1]. Furthermore, the Advisory Council for Aeronautics Research in Europe (ACARE) plans to reduce CO_2_ emissions by 75% and nitrogen oxide emissions NO_x_ by 90% by 2050 compared to the levels in 2000 [2]. However, in contrast to the automotive industry, the high energy density required in aviation prevents a fundamental change in the drive concept [3]. Currently, nickel-based superalloys with a density of up to 8.9 g/cm³ are used in the hot areas of aircraft turbines [4]. Oxide fiber composites (OFCs) with a density of 2.4 to 2.9 g/cm³ have several advantages over the nickel-based superalloys used as the standard material in this temperature range. These include a weight advantage of up to 73% as well as better thermomechanical properties with service lives of over 20,000 h for industrial applications [5,6,7]. Due to their chemical resistance and inherent oxidation stability, even at operating temperatures of over 1000 °C in corrosive atmospheres, OFCs are potential key elements for developments in energy conversion and transport technology [6].

In order to adjust the damage-tolerant fracture behavior, materials research on OFCs primarily pursues two research approaches. In the first approach, an attempt is made to selectively weaken the bond between the reinforcing fiber and the matrix by means of a coating. However, the application of fiber coatings is cost-intesive, as is the fairly dense matrix, which often requires a high number of reinfiltrations with preceramic polymers, in order to reach its desired density. In the second approach, research is being conducted on the generation of fracture-tolerant and fault-tolerant material behavior of composite ceramics with the concept of a porous matrix. However, the mechanical properties are lower in matrix-dominated, complex and interlaminar loading situations compared to composites with a fiber coating. An intrinsic weakness of OFC components with a weak matrix is the high susceptibility to shear. The interlaminar shear strength (ILSS) has only a fraction of the flexural strength, therefore limiting the range of applications. This damage tolerance leads to lower mechanical properties compared to monolithic ceramics [7,8,9,10]. The deficit of the porous matrix is to be eliminated by a three-dimensional reinforcement structure suitable for shear loading.

**Figure 1:**
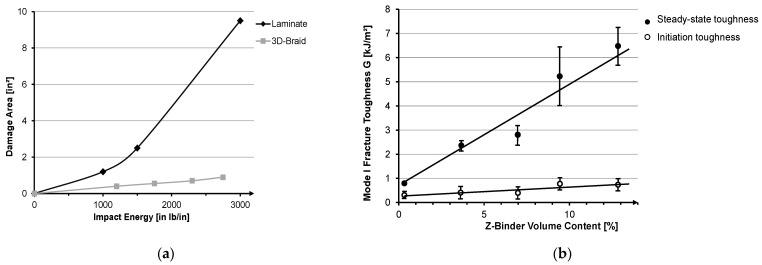
Energy required to initiate and propagate damage of 3D braids (**a**) [11]. Reprinted with permission from [11]. Copyright 2017 Elsevier. Effect of Z-binder yarn content on the initiation and steady-state fracture toughness values (**b**) [13]. Reprinted with permission from [13]. Copyright 2016 Elsevier.

Considering a carbon-fiber-reinforced polyether ether ketone (PEEK) composite (Figure 1a), the energy required for damage initiation and propagation is significantly higher for composites with a 3D braided structure than for 2D braids and fabrics [11,12]. Fishpool et al. show that Z-binder yarns in 3D woven composites increase the mode II fracture toughness up to three times compared to a 2D woven laminate [13,14]. Further research regarding the correlation between Z-binder volume content and mode I fracture toughness shows a quasi-linear increase (Figure 1b). The maximum value of the steady-state fracture toughness is reached with the highest Z-binder content (13.2%) and is almost eight times higher than for woven 2D laminates [13].

So far, OFCs have two-dimensional reinforcements, produced either by winding technology from rovings or by hand lay-up processes from woven fabrics [15]. The maximum flexural strength of fabric-reinforced OFCs is 280 MPa to 400 MPa with elongations at break ranging from 0.2% to 0.5% [7]. The average interlaminar shear strength is 11 MPa to 19 MPa, depending on the used fibers and matrix system [16]. Commercially available reinforcements are exclusively 2D with no fibers in the Z-direction orientation, resulting in inadequate mechanical properties under interlaminar loading. A solution approach is the use of near net shape preforms with 3D reinforcement. Worldwide, Nextel^TM^610 (alumina) or Nextel^TM^720 (mullite/alumina) fibers are almost exclusively used for OFC reinforcement [17]. Fibers from other manufacturers or other Nextel grades are hardly ever used to reinforce ceramics due to insufficient temperature resistance, mechanical properties and/or processability [18,19].

## 2. Materials and Methods

The braiding process chain is divided into the winding process and braiding. The manufactured preforms are subsequently infiltrated with a ceramic slurry to prepare the OFC. The bobbin is the central component of any braiding machine and essential for storing the yarn, maintaining the yarn tension and for low-damage yarn processing. Compared to radial and circular braiding, the yarn compensation length in the yarn drop is much greater in a 3D braiding process. For the manufacturing process of 3D braiding, bobbins are newly developed which are suitable for processing brittle ceramic oxide fibers. A comparison of Nextel^TM^610 (3M Corporation, Maplewood, MN, USA) fibers with 10,000 den versus fibers with 1500 den shows a cost advantage for fibers with 10,000 den, with only minimal differences in mechanical properties [20].

**Figure 2:**
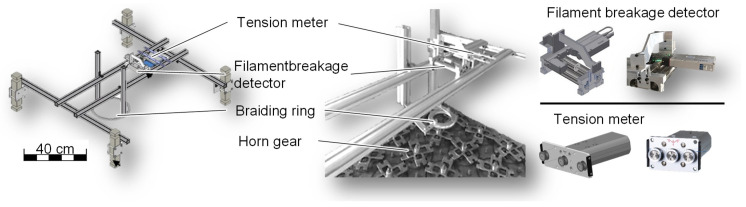
Yarn tension meter and filament breakage rate meter integrated into the 3D braiding process.

After the conceptual investigation of possible bobbin variants, a technical analysis is carried out. The highest potential bobbin is developed within the framework of VDI 2221. The VDI 2221 is the standard work for designers or product developers and covers the fundamentals of methodical development of all types of technical products and product systems. Tribological investigations in the 3D braiding process are used to quantify the fiber stress. To quantify fiber wear, the fiber tension, i.e., the value responsible for a uniform preform, and the filament breakage rate are recorded (Figure 2).

The fiber wear is characterized on the basis of the filament breakage rate. For this purpose, protruding filaments are detected by sensors under the light barrier principle. The measured filament breaks per minute are related to the same speed so that all evaluations, regarding the number of filament breaks per minute, are converted to the take-off speed of 1.25 m/min. One test cycle lasts 5 min. All tribological tests are performed exclusively with Nextel^TM^610 10,000 den fibers.

Subsequently, the braiding process is modified regarding fiber-friendly processing and a preform is produced. After preform production, the fiber semifinished products are further processed into OFCs. For this purpose, the preforms are first desized for 2 h at 700 °C in air and then further processed into OFCs via a two-stage vacuum infiltration process [21] or a prepreg process [22]. The solid content of the aqueous slurries comprises 70 wt% alumina with a d50 of 0.7 µm (CT 3000 SG, Almatis, OYAK, Ankara, Turkey), 5 wt% alumina with a d50 of 0.1 µm (TM-DAR, Taimei Chemicals, Nagano, Japan) and 25 wt% 3YSZ (TZ-3Y-E, Tosoh, Tokyo, Japan) [22]. As a dispersing agent, 1 wt% Sokalan PA 15 (BASF, Ludwigshafen, Germany) was used. While the solid content of the used slurries differs for each method, the glycerol (99.5% AnalaR NORMAPUR^®^, VWR, Darmstadt, Germany) content in regard to the solid content remains constant at 26 wt%. The slurries are prepared by ball milling with 3 mm zirconia beads in a drum mill, until the mean and maximum particle size is below 1 µm or 5 µm, respectively [21,22]. The bimodal particle size distribution together with different sintering activities of the powders leads to a low shrinkage of the matrix, thereby preventing the detachment of fibers from the matrix during sintering. During the two-stage vacuum infiltration process, the fiber preforms are first pre-infiltrated with a low-viscosity slurry (30 wt% solid content) at normal pressure, in order to build up the intra-bundle matrix. Subsequently, the inter-bundle matrix is formed by infiltration in a vacuum setup, with a slurry with higher viscosity (65 wt% solid content). After an initial drying step at room temperature for 1 h, the vacuum setup is placed on a heating plate at 70 °C for 2 h in order to obtain a self-supporting green body [21]. For the prepreg process, the fiber preforms are infiltrated with a slurry with 67 wt% solid content by means of a brush. By conditioning them in a climate chamber (305SB/+10 IU, Weiss, Reiskirchen, Germany) at 53%r.H/25 °C, the water content and thereby the tack of the prepregs is adjusted, rendering them suitable for lamination using a cold roll laminator. After lamination, the samples are dried for 2 h at 70 °C, followed by 12 h at 100 °C [22]. For both processing steps, the OFCs are obtained by sintering the self-supporting ceramic green bodies for 2 h at 1225 °C in air. Braided 2D and 3D preforms consist of α-alumina Nextel^TM^610 (3M Corporation, Maplewood, MN, USA) fiber bundles with 10,000 den, whereas the used 8HS fabric DF-19 comprises 3000 den fiber bundles. For each fabric-reinforced OFC, four fabric layers were stacked, while the braid reinforcements were not stacked but used as a single reinforcement for each OFC.

Samples for mechanical testing were prepared using a water-cooled diamond wire saw Type 6234 (well Diamantdrahtsägen GmbH, Mannheim, Germany). The interlaminar shear strength (ILSS) of an OFC with reinforcements consisting of either 2D fabrics, 2D braids or 3D braids is determined using 3-point short bending tests according to DIN EN 658-5 with a support span of 15 mm and sample sizes of 25 × 10 mm^2^. Furthermore, the bending strength of the OFCs is determined by a 3-point bending test according to DIN EN 658-3 with a support span of 60 mm and sample sizes of 70 × 10 mm^2^. The used traverse speed for both test methods is 1 mm/min. For every test method, at least five samples of each OFC are tested.

The relative macroporosity of the OFC was calculated based on the density and porosity measured by the Archimedes principle according to [22].

## 3. Results and Discussion

The results and the discussion are divided into process chain modification and design in the context of textile development and into infiltration and testing in the context of fiber composite ceramic development.

### 3.1. Results and Discussion—Textile Development

The braiding process chain is divided into the process chain stages of winding and of braiding. Both stages are examined and improved.

#### 3.1.1. Winding Process Design

Hairiness caused during the winding process increases the damage in the subsequent process steps, since filament breakage increases friction. When looking at the residual tensile strength of good and poorly spooled fibers, a far greater increase in fiber breakage is seen in poorly spooled fibers, leading to pre-braiding and ring formation. Therefore, the rewinding process plays a significant role in avoiding or reducing fiber breakage along the process chain. The rewinding is carried out on a semi-automatic winding machine, type SP 280, and a pay-off creel, GU 16 (Herzog GmbH, Oldenburg, Germany). The parameters that can be varied are the winding speed (20 m/min, 40 m/min or 60 m/min), the winding type (parallel or cross-winding), the yarn brake and the creel braking force (2 N). 

**Figure 3:**
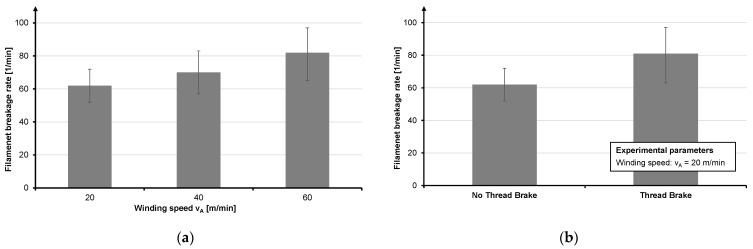
Influence of winding speed (**a**) and yarn brake (**b**) on filament damage.

From the analysis of the winding process (Figure 3), it can be seen that the fiber damage decreases with decreasing speed. A take-off speed of 20 m/min leads to the lowest filament breakage. The creel brake is set to 2 N and no yarn brake is used, since this causes severe yarn damage because of friction. Cross-winding should be chosen as the winding method as this ensures a lower speed increase when the yarn bobbin empties. Moreover, an increase in the speed is accompanied by yarn tensile force, which should be avoided when processing highly brittle fibers [23].

#### 3.1.2. Bobbin Development

**Figure 4:**
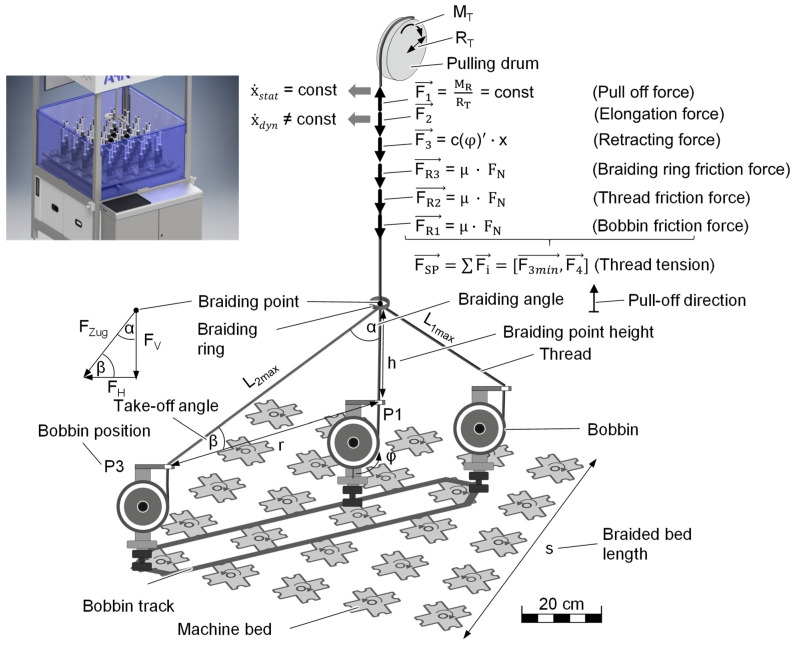
Kinematics and kinetics on the braided yarn.

In 3D braiding, maintaining the yarn tension throughout the process is necessary to produce a uniform braid. The requirements for the bobbin are a sufficient compensation length to compensate for the yarn drop, the constant yarn tension required for a uniform preform structure, low filament breakage to ensure high mechanical properties and in general a stable process. For this purpose, the influences on the bobbin responsible for tension peaks and the resulting filament breakage are identified, and kinetic relationships are derived [24]. The filament tension of the braided yarn depends on the forces acting on the yarn (Figure 4).

The yarn tension force can be defined via the sum of the applied forces:

(1) $$\vec{F_{\mathrm{SP}}}=\vec{F_{1}}-\vec{F_{2}}-\vec{F_{3}}-\vec{F_{R1}}-\vec{F_{R2}}-\vec{F_{R3}}$$

The take-off force, F1, is generated by the rotating take-off drum, which pulls the braided thread upwards. Only when the yarn tension force (F_SP_) exceeds the holding force (F_SPMax_) does a movement of the shaft in the bobbin cause the spring to be wound back inside the bobbin. The length-changing force (F_2_) is a result of the movement of the bobbin on the machine bed, which continuously changes the length of the braided yarn. The yarn length increases when the bobbin is moved away from the center of the machine bed. In the opposite case, when the bobbin is moved towards the center of the machine bed, the length of the braiding thread decreases again. The compensation of the yarn length is done by the pull-back force (F_3_), which ensures that the braiding yarn is wound back onto the bobbin. The retraction force, F_3_, corresponds to the spring force and can thus be determined by the choice of spring parameters. Furthermore, there are frictional forces acting on the yarn tension force. On the one hand, this is the friction of the braided yarn on the braiding ring (F_R2_) and on the braiding eye (F_R3_). On the other hand, there is the frictional force between crossing threads (F_R1_). 

#### 3.1.3. Fiber Damage Influences on the Bobbin

According to Rosenbaum, most of the fiber damage in a vertically supported bobbin occurs during take-off from the supply bobbin, due to the yarns being diverted in the longitudinal direction of the bobbin [25]. The filament thereby rubs proportionally across the wound fibers. To avoid this, a 90° angle would be ideal during take-off from the bobbin (Table 1). To achieve an approximately tangential take-off, the use of a horizontally mounted bobbin with a high thread eye is recommended. In any case, the relationship between the width of the bobbin or the spooling and the distance of the take-off point should be negligible. A solution for vertically supported bobbins is offered by an electronically controlled bobbin with a self-adjusting sensor-controlled first deflector according to Van Reden [26]. Partial solutions for vertically supported bobbins are offered by baffles, which cause less yarn wear due to more suitable friction pairing. Another solution is to use slider bobbins with a movable spring-loaded first-deflection roll. In any case, the integrity of the sizing is essential to achieve low filament breakage.

Another factor influencing gentle fiber processing is filament breakage due to friction as a result of the deflection at yarn guiding elements. As a result of contamination of yarn guiding elements, fluctuations in yarn tension of up to 25% can occur [25]. Consequently, clean and unworn guiding elements are essential. In the bobbin, the yarn is guided by rigid or rollable deflection elements (Table 1). In the case of rigid elements with relative movement between the friction partners, the friction force is calculated using the Euler–Eytelwein formula (Equation (4) in Table 1). Here, $F_{\mathrm{After}}$ is the tensile force in the direction of the braiding point and $F_{\mathrm{Before}}$ is the tensile force in the direction of the thread spool. From this equation, it follows that reducing the wrap angle, γ, leads to a reduction in the frictional force acting on the yarn. In general, with regard to yarn-protecting processing, a horizontal bobbin with a minimum number of yarn guiding elements should be preferred to a vertical bobbin. Moreover, two factors are influenced by the choice of deflection radius. On the one hand, the curvature of the yarn at the contact point is determined, which can be critical for brittle materials, because excessive bending leads to yarn breakage. On the other hand, the deflection of the tensioned yarn creates forces between the yarn and the guide element. The length over which these forces can be distributed is determined by the variation of the deflection radius at a constant wrap angle. Thereby, the friction reduction must be achieved by suitable material pairing and geometry. Using deflection rollers with no or only a small amount of relative movement is better, as these cause a smaller increase in the yarn force [27]. However, due to the abrasive nature of ceramic fibers, the main focus is on preventing yarn guide wear.

In addition, yarn breakage is caused by mass inertia, bearing and gear-related frictional resistance, whose influences can be calculated via a moment equilibrium at the pivot point (Equation (5) in Table 1). The fiber force, $F_{\mathrm{Fiber}}$, results in a rigid state from the quotient of the applied torque (M_F_) of the rotational compensation mechanism, e.g., spiral spring, to the bobbin radius (r). In a dynamic state, during unwinding, the quotient of inertia ($J_{S}$) and coil radius (r) multiplied by the angular acceleration of the filament coil, ${\ddot{\varphi }}_{S}$, is added. Furthermore, the thread is additionally loaded by the frictional resistance in bearings and gears.

Essential for the fiber damage at the bobbin is also the raveling of fibers during the unwinding process (Table 1). According to Mierzwa et al., the formation of fiber rings when unwinding the yarn from the bobbin during the braiding process is the main cause of process-critical braiding faults [28]. This defect pattern is typical for untwisted multifilament yarns and rovings, as used for the reinforcement of ceramic matrix composites. In this case, individual filaments or filament bundles detach from the main strand, thus the yarns are not unwound and remain as a ring-shaped filament accumulation on the respective bobbin [29]. This initially increases the tension in the unwound yarn due to the increasing friction. As a result, the braid contracts unevenly and gaps appear between the braided filaments, leading to a reduction in preform quality and scrap. As the ring formation progresses, the yarn friction can increase to such an extent that the yarn concerned is torn [29,30]. This phenomenon can be prevented by avoiding fiber damage in the winding process and by using large radii when winding back onto the bobbin.

#### 3.1.4. Concept

**Figure 5:**
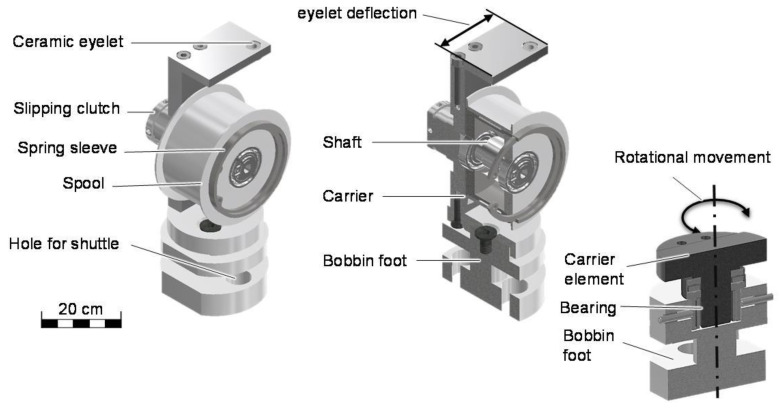
3D bobbin modifications for better processing of ceramic fibers.

The conception is carried out by taking into account the influencing factors, which damage the yarn, and the state of the art regarding existing patents and publications of bobbin lace. To reduce complexity, only purely mechanical solutions for yarn compensation are considered. Motorized concepts are not considered due to the demanding and damage-prone control system as well as the high development risk. Taking into account the four influencing factors causing fiber damage (Table 1), a horizontally mounted bobbin is considered the most promising solution. In contrast to the patents [31] and [32], a toothed gear between the bobbin and the balancing mechanism is not used due to the aim of gentler processing. Due to the large radius, the compensation mechanism will be relocated into the bobbin [33,34,35,36,37]. The following purely mechanical compensation mechanisms are conceivable: a rolling spring with a slip clutch, according to [35], and a spiral spring with a slip clutch, according to [36] and [33]. A torsion spring according to [34] is considered insufficient in terms of compensation length. A magnetic brake or eddy current brake according to [34] and [38] is desirable. A cost–benefit analysis shows that a slipping clutch is a cost-efficient alternative for laboratory operation. Taking into account the above-mentioned aspects, a bobbin is developed according to Figure 5, which additionally has a deflected ceramic eyelet and has a rotational bobbin foot.

#### 3.1.5. Component Design

Due to the high standard deviation in the procedural measurement of the yarn breakage rate in the braiding process, the tension level and the change in tension are considered to be the cause of the yarn breakage, similar to Büsgen, Rosiepen and Schneider [24,27,39]. This assumption is particularly plausible, as the simple construction of the bobbin lace base with the minimization of thread guiding elements reduces the influence of friction elements.

**Figure 6:**
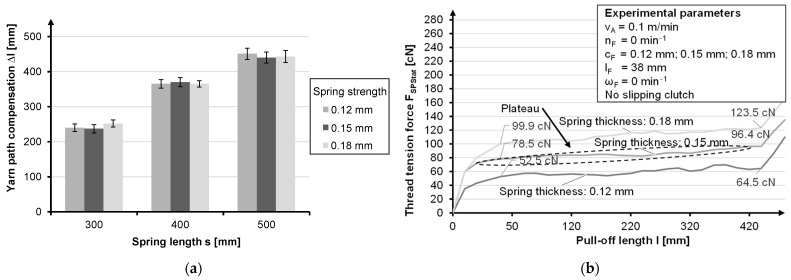
Influence of spring length on compensation length (**a**) and influence of spring thickness on thread retraction force (**b**).

Stainless steel spring strips with a width of 12.7 mm and with a spring thickness of 0.12 mm, 0.15 mm and 0.18 mm are used as return springs. In order to maintain the yarn tension over the entire braiding process even at maximum deflection and to deflect it only at the reversal point, the spring length is selected accordingly (Figure 6a). With regard to the compensation travel, the choice of spring thickness is not decisive. Thicker springs only mean a higher thread force on the plate and less material in the spring barrel (Figure 6b). Regardless of the spring thickness, all springs show a spring characteristic with an approximately 20% increase in the yarn tension force (F_SPStat_) under a quasi-static load at minimum take-off speed without bobbin motion and without a slipping clutch.

**Figure 7:**
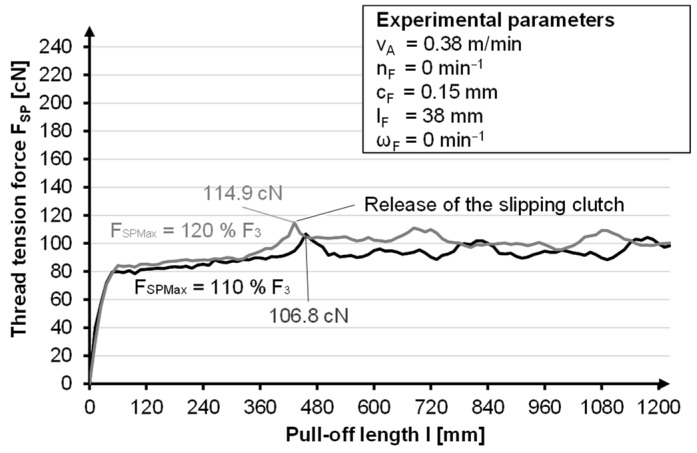
Representation of the yarn tension force as a function of the slipping clutch.

With the help of Mädler slip clutches type A (MÄDLER GmbH, Stuttgart, Germany) a torque range of 2.4 Ncm to 53.8 Ncm can be generated with two friction discs and of 7.8 Ncm to 132.4 Ncm with six friction discs. This means that with a bobbin radius of 30 mm, holding forces (F_SPMax_) of 0.8 N to 44.13 N can be achieved. As soon as the holding force is exceeded, the shaft rotates and the spiral spring turns back, which means a drop in yarn tension (Figure 7).

At the beginning, a linear increase in the thread tension force F_SP_, can be seen. This increase is a result of the spring characteristics. As soon as the maximum spring force, F_3_, is reached, the thread tension force, F_SP_, increases sharply. When a holding force, F_SPMax_, of 110% (120%) of the spring force is selected, the maximum thread tension force, F_SP_, is 106.8 cN (114.9 cN). As soon as the thread tension force exceeds the holding force, F_SPMax_, of the slipping clutch, the slipping clutch is released. The shaft can now rotate, and so the yarn tension force decreases again. The further course of the thread tension force shows that it remains constant with a deviation of approximately ± 10 cN.

The measurement of the thread tension force, F_SP_, shows that a holding force, F_SPMax_, of 110% of the spring force, F_3_, is sufficient to ensure the mechanism of the slip clutch and to achieve an approximately constant thread tension. A higher holding force causes a higher tension peak, which leads to an additional mechanical load on the thread and should therefore be avoided.

**Figure 8:**
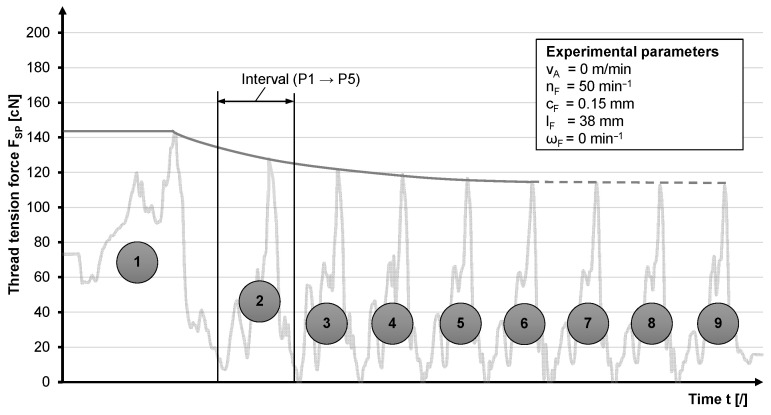
Settling time of a bobbin with a horn gear speed of 50 min^−1^.

Within the scope of the statistical design of experiments, the influence on the yarn tension is investigated as a function of the take-off speed (v_A_), horn gear speed (n_F_), spring thickness (c_F_), yarn eye deflection (l_F_) and rotatability of the bobbin foot (ω_F_) variables. Two values are selected for each variable (Table 2). The tests show a regressive amplitude curve with different settling times due to the mechanical shock during start-up and the inertia of the system. The greatest influence on the settling times is exerted by the horn gear speed. For a horn gear speed of 3.75 min^−1^ the settling time is approximately three to four cycles, and for 50 min^−1^ it is approximately six to eight cycles. The latter is illustrated in Figure 8. With a spring thickness of 0.12 mm and a horn gear speed of 50 min^−1^, the low stiffness leads to the prevention of a transient due to a too slow and too weak return and a resulting loss of preload force. Other parameters, such as the yarn eye deflection and the rotational pivot of the bobbin, have no effect on the settling time. The take-off speed shows no effect on the settling time of the process.

In order to obtain comparable values, the thread tensions in the steady state after eight cycles for 50 min^−1^ and four cycles for 3.75 min^−1^ are compared as a function of the horn gear speed. During start-up, the slip clutch release force is exceeded in the form of a peak with values of up to 140 cN. This delayed reaction usually occurs only initially and is interpreted as a breakaway force in the mixed friction contact due to downtime (Figure 8).

**Figure 9:**
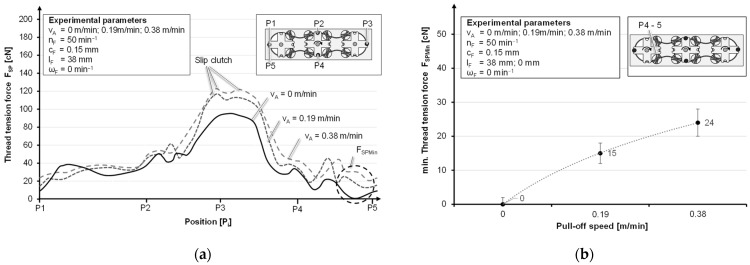
Thread tension curves at different haul-off speeds (**a**) and averaged minimum thread tension at different haul-off speeds (**b**).

Increasing the take-off speed from 0 m/min via the intermediate value 0.19 m/min to 0.38 m/min leads to a steady increase in the minimum, whereby the maximum is limited by the slipping clutch. The averaging of the time intervals eight to twelve of each test series shows an increase of 15 cN on average for the yarn tensile force when the take-off speed is increased from 0 m/min to 0.19 m/min. The increase in the take-off speed from 0.19 m/min to 0.38 m/min leads to a further increase of 9 cN (Figure 9b). The different dynamics in the system due to an increase in the take-off and bobbin movement and the inertia result in a release of the slipping clutch at higher thread tension forces, lying between 115 cN and 125 cN (Figure 9a).

**Figure 10:**
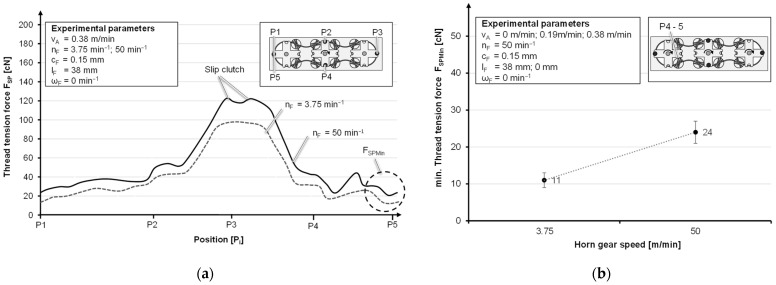
Thread tension force curves at different horn gear speeds (**a**) and averaged minimum thread tension force at different horn gear speeds (**b**).

When moving without thread take-off, the load is low enough; as a result, the slipping clutch does not have to be triggered. The loss of preload brings more dynamics into the system until a certain value of the tension is reached again. The influence of the horn gear speed, which is much higher than the take-off speed, drastically increases the dynamics in the system. Further, this leads to an increase in the minimum yarn tension force from 11 cN to 24 cN when changing from 3.75 min^−1^ to 50 min^−1^ (Figure 10b). The maximal tension force is increased until the slipping clutch is triggered (Figure 10a).

While investigating the influence of the yarn eye position on the bobbin and the radial rotary bearing of the bobbin foot, the effect on the maximum yarn tension is considered and the slip clutch is locked in place. 

**Figure 11:**
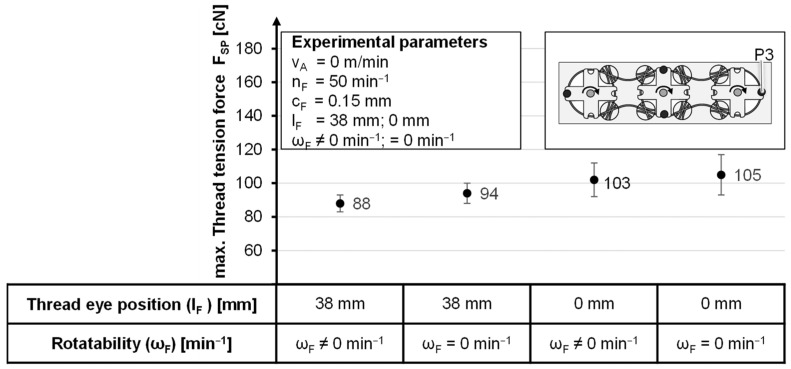
Maximum yarn tension force as a function of the yarn eye position and the rotatability of the dished foot.

When the thread eye is shifted to the center of the bobbin, an increase in the maximum thread tension force is shown compared to the deflection of 38 mm. This is due to the 45° larger wrap angle at the thread eye, regardless of the bobbin position. Even an integration of the rotating foot shows only minimal improvement, as the bobbin hardly aligns. This is because the alignment of the bobbin generates a torque around the axis of rotation of the lever arm. The torque is created by the displacement of the thread eye (Figure 4). As a result of the favorable alignment, the rotary motion leads to a minimization of the deflection and reduction in the axial sliding movement at the eyelet, allowing the maximum thread tension force to decrease by up to 6 cN (Figure 11). The rotating foot shows about 15% better values at horn gear speeds of 3.75 min^−1^ than at 50 min^−1^, as a slight overshoot can be seen. However, due to the significantly higher productivity, a horn gear speed of 50 min^−1^ is negligible, with a minimal difference of 2 cN (Figure 11).

Table 2 summarizes the effects of the influencing factors take-off speed, horn gear speed, spring thickness, thread eye deflection, rotatability of the dished foot on the maximum and minimum thread tension force as well as the transient length when changing the value from the lower to the upper value.

#### 3.1.6. Braiding Ring

A “natural braiding point” is formed as a function of the material used and the process parameters, the most important in this context being yarn tension, braiding ring height, braiding ring diameter, horn gear speed, fiber hairiness, number of fibers, loading and the use of a vibration unit. The more friction there is between the yarns and the slower the yarns move vertically, the lower the natural braiding point. This affects the braiding angle (α) as well as the take-off angle (β). According to Engels, a minimum take-off angle of 27° is recommended to avoid collisions [23]. The braiding point height (h) (Figure 4) is calculated as a multiple of the bisected braid bed diagonal (r). The height which indicates the maximum horizontal distance from the braiding point is calculated from the braid bed side length (s).

(6) h=n·r

(7) $$r=\frac{s}{\sqrt{2}}$$

The ratio of the braiding point height (h) and the half diagonal (r) results in a braiding angle (α), with its characteristic regressive course, and a take-off angle (β) with a degressive course.

(8) $$\frac{r}{h}=\tan\left(\alpha \right)\;$$

(9) $$n=\frac{h}{r}=\tan\left(\beta \right)\;$$

Using the braiding angle, the yarn tension force (F_tension_) can be divided into a horizontal force component (F_H_) and a vertical force component (F_V_), whereby the horizontal force shows a regressive and the vertical force a degressive force curve.

(10) $$F_{V}=F_{Zug}\cdot \cos\left(\alpha \right)$$

(11) $$F_{H}=F_{Zug}\cdot \sin\left(\alpha \right)$$

Large horizontal forces cause compaction of the braid, which leads to a lower fiber volume content in the fiber composite and tends to lower mechanical properties. Due to the slipping of the yarns at a low braiding point, the yarns are tendentially pressed into one plane. This increases fiber–fiber friction, which in turn increases filament breakage. The optimum would be an infinitely distant braiding point where all fibers are parallel. In the machine used, a minimal n-ratio of 0.51 (for a braiding point height of 332 mm) and a maximal ratio of 1.7 (for a braiding point height of 1100 mm) can be set. Thus, a maximum compensation length between 17.8 cm and 37.8 cm is required, depending on the height of the braiding point.

(12) $$\Delta x=(\sqrt{1+\frac{1}{n^{2}}}-1)\cdot n\cdot r$$

If the braiding ring is not used, the natural braiding point will nevertheless form but at a slightly larger height. Ideally, the natural braiding point is selected so that the braiding angle is only set by the horn gear speed and the take-off speed, and there is practically no braiding ring contact.

When investigating fiber stress and damage on the braiding ring, one-factor-at-a-time (OFAT) is used as a guidance to narrow down the test volume to find the best values regarding the friction body diameter and the friction body material. Then, design of experiment (DoE) is used to reproduce the interrelationships between several variables. The process parameters (Table 3) are investigated with respect to the influence on the friction coefficient and filament breakage rate. An evaluation of the dished head on the trobological test bench is only possible by continuous peeling, without consideration of the filament fall. The test bench consists of two static thread tension meters; the first sensor is placed between the bobbin and braiding ring and the second after the braiding ring. This was possible because there was no movement of the bobbin. The filament breakage rate is determined after the braiding ring immediate in front of the thread tension meter. In order to exclude roughness influences, guide plate models with similar averaged roughness values are used. 

**Figure 12:**
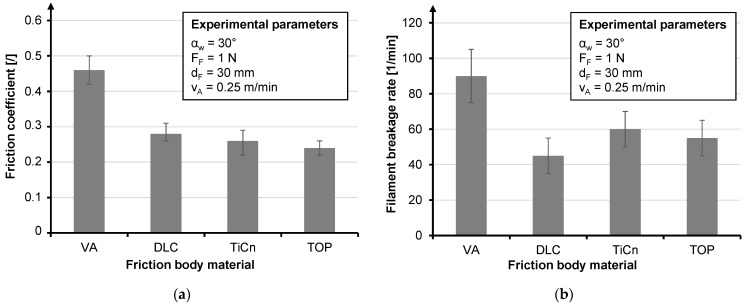
Influence of base materials regarding the friction coefficient (**a**) and influence of base materials regarding the filament breakage rate (**b**).

An excerpt from the tests, which shows the most concise results, is given. Due to the high braid ring position, a wrap angle (α_W_) of 30° is assumed for all tests. Since the data set is an extract from a larger data set, it should be noted for the sake of comparability that the values for the filament breakage rate refer to a haul-off speed of 1.25 m/min which represents an industrial production speed. The influence of base materials is investigated by using stainless steel, which is coated with diamond-like carbon (DLC) and titanium carbonitride (TiCN) of CeWOTec GmbH, Chemnitz, Germany and Topocrom No. 131 (TOP) of Topocrom GmbH, Stockach, Germany. The DLC layer causes the lowest filament breakage rate of all friction bodies (Figure 12b), but its coefficient of friction was not the lowest (Figure 12a).

**Figure 13:**
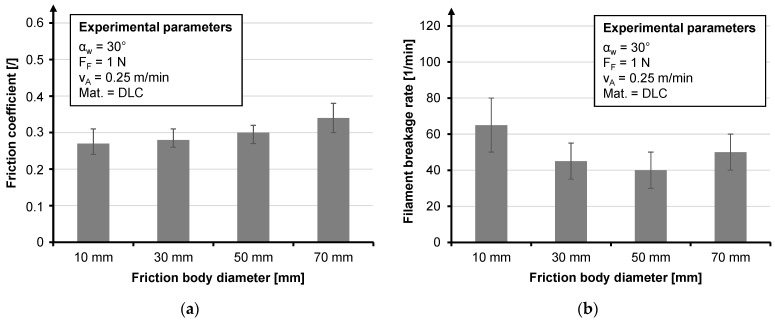
Influence of the friction body diameter regarding the friction coefficient (**a**) and influence of the friction body diameter regarding the filament breakage rate (**b**).

The tests on the braiding ring indicated a slight increase in the coefficient of friction with an increase in the diameter of the friction body (Figure 13a), and a decrease in the filament breakage rate between 10 mm and 50 mm (Figure 13a). It is noticeable that between 50 mm and 70 mm the filament breakage rate increases again; this is explained by the longer contact distance, whereby the lower bending resulting from the lower fiber curvature has less influence on the filament breakage rate. With respect to the coefficient of friction, the results coincided with those of Lünenschloß for polymer fibers [40]. Due to the drop in filament breakage rate between 10 mm and 30 mm, a diameter of at least 30 mm is recommended.

**Figure 14:**
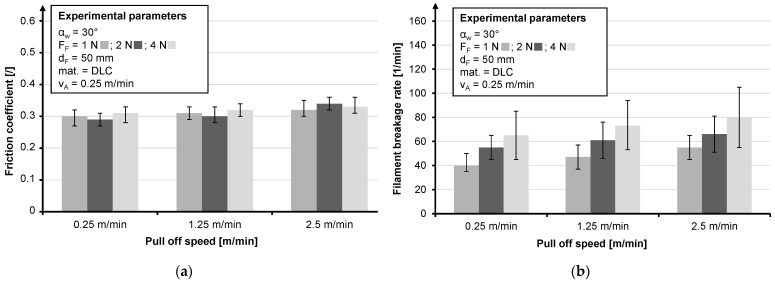
Influence of the pull-off speed and the yarn tension force regarding the friction coefficient (**a**) and influence of the pull-off speed and the yarn tension force regarding the filament breakage rate (**b**).

For the combination of an increase in fiber tension force and pull-off speed, the results show an increase in pre-stress, which causes an increase in the filament breakage rate (Figure 14b). The best results are obtained for a fiber force of 1 N. Increasing the pull-off speed caused a minimal increase in the coefficient of friction (Figure 14a). The combination of high force and high pull-off speed is particularly critical.

It is noticeable that, when increasing the pull-off speed and yarn tension force, increasingly more broken filaments lie in the machine bed. Mierzwa et al. describe in [28] a similar effect for carbon fibers, where above a certain pull-off speed and yarn tension force the broken fibers do not remain in the fiber and can be detected with the laser detector. It is assumed that this effect is responsible for the growing standard deviation.

The basic recommendation is to increase the bending radii of the filament contacting elements to at least 30 mm and to reduce the fiber force to less than 1 N and to a low processing speed. For successful processing, the avoidance of self-reinforcing damage accumulation is essential. Due to this, a non-constant winding process without yarn brakes is also recommended, and active motor-driven unwinding is to be preferred over tangential unwinding. The measured, averaged roughness depth values, R_Z_, moved in an interval between 5.071 μm and 7.185 μm for the guide sheet models (⌀50 mm and ⌀70 mm). An influence can be ruled out due to the small difference below the 40 μm limit [24]. A braiding ring should be avoided whenever possible because of the additional contact, and a natural braiding point should be preferred for braiding. If a large braiding angle is required, a braiding ring with a torus radius of at least 30 mm is recommended to reduce the bending load. To increase wear resistance and reduce roughness, a DLC coating is suggested. To reduce the horizontal force component of the yarn tension in the bobbin, a yarn force of 1 N is proposed.

**Figure 15:**
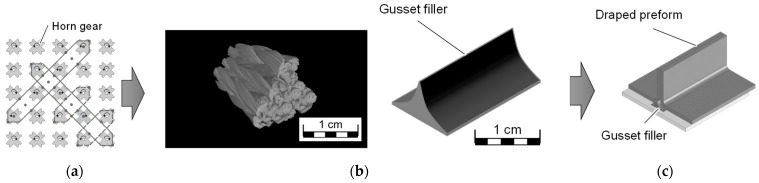
Braiding pattern of a gusset filler (**a**); CT image and sketch of a gusset filler (**b**); and gusset filler in draped preform (**c**).

Three-dimensional braiding technology contributes to an increase in the delamination resistance of porous OFCs, which in the long term enables an expansion of the application range to higher mechanical loads. Possible applications of 3D braided ceramic structures are gusset fillers for large-area T, I, Z, L and Ω profiles, since the structures have already been tested on a laboratory scale. Gusset fillers are essential for large, draped structures, as these are used to compensate for edges that occur during draping (Figure 15b). To produce a gusset filler, 14 braided yarns and 8 standing yarns are used. By combining braided threads with 10,000 den and standing threads with 20,000 den, a triangular structure can be achieved (Figure 15a).

Due to the geometric design possibilities of the 3D braiding process, complex profiles such as turbine blades can also be realized. The size of the profiles can either be increased by using a larger braiding bed with more bobbins, fibers with a higher titer (e.g., 20,000 den) or already braided, plied or twisted yarns.

### 3.2. Results and Discussion—Fiber Composite Ceramics

The braiding process is modified regarding fiber-friendly processing, and a preform is produced. The finished preforms are infiltrated with a low-shrinkage slurry with different fabrication processes [21,22]. The interlaminar shear strength (ILSS) is determined using 3-point short bending tests according to DIN EN 658-5. Furthermore, the bending strength of OFC with 2D fabrics, 2D braids and 3D braids reinforced with Nextel^TM^610 10,000 den fibers is determined by a three-point bending test according to DIN EN 658-3. At least five specimens of every OFC were tested. The OFCs with fabric reinforcement comprise four layers of 8HS fabric DF-19 with 3000 den fiber bundles. For the production of 2D braids, braided tubes produced by radial braiding are flattened, creating a slight Z-reinforcement on the side with a Z-fiber content of 1.5%. As part of the process design, the braiding angle (30°, 45° or 60°), spring strength (100 g or 1 N as well as 200 g or 2 N) are varied with and without the use of a vibrator and evaluated with respect to ILSS (Table 3). In the center of the specimen, the braid exhibits unreinforced areas generated by the springback of the Z-reinforcement at the edges. For this test series, all samples are manufactured using the vacuum infiltration process, because there the drying is performed in the vacuum setup, whereas in the prepreg process springback is highly probable after the lamination step due to the lack of external pressure. 

Lower braiding angles show higher interlaminar strengths due to the lower angle between the fibers and the loading direction. In addition, resistance to compaction decreases at lower braiding angles, resulting in higher fiber volume contents. The different spring tensions do not affect the interlaminar shear strength, but higher spring tensions result in lower fiber volume content due to higher resistance to compaction. In addition, the higher spring tension leads to increased fiber damage. This is evident from the hairiness of the fiber bundles, i.e., the broken individual fibers protruding from the bundle. The vibrator shows no clear influence on the mechanical properties of the OFCs, as the differences between specimens with “vibrator on” or “vibrator off” are comparable within the standard deviation. However, the mechanical values with the “vibrator off” setting tend to be somewhat higher than those with “vibrator on”. The ILSS is in the range of that of fabric-reinforced OFCs (12.4 ± 2.4 MPa) with the same matrix system (Table 4). From the investigations, a braiding angle of 30°, 100 g spring tension and the “vibrator off” setting are identified as advantageous for the braids described.

**Figure 16:**
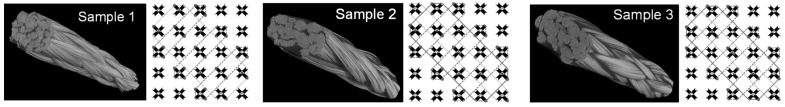
Three-dimensional braided sample 1, sample 2 and sample 3.

In the 3D braiding process, three specimens, each with 28 braiding threads with different Z-fiber content, are produced. The samples are varied in terms of Z-fiber content at a superficial braiding angle of 30° predetermined by the preform structure. The different Z-fiber contents are 7.2% (sample one), 10.6% (sample two) and 13.1% (sample three). Figure 16 shows CT images of the samples. The Z-fiber content is determined by a dynamic simulation in which bobbin speed, number of threads, motion pattern, twist, pull-off speed and filament number are taken into account. The multifilament yarn is represented by 12 filaments, forming an octagon. The acting forces are represented by a displacement algorithm which leads to compaction. The boundary conditions are fitted to the CT image by the fiber angle, regarding the angular progression of the fibers along corresponding planes. The OFCs are prepared by the prepreg process [22], since preliminary tests with the vacuum infiltration process showed a very rough surface of the samples, therefore rendering these unsuitable for testing. The samples prepared by the prepreg process do not exhibit this problem. In addition, there is no danger of springback as for the 2D braids, as this is prevented by the Z-fiber content across the entire cross-section.

**Figure 17:**
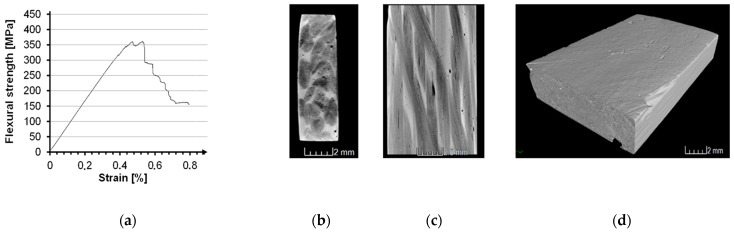
Representative bending stress–strain diagram of a 3D braided OFC (**a**), a µ-CT cross-section of a 3D braided OFC (**b**), a µ-CT plan view of a 3D braided OFC (**c**) and a µ-CT view of a 3D braided OFC (**d**), each of sample 3.

Investigations of the microstructure by µ-CT (Figure 17c) show a small amount of macroporosity. Due to the spherical pore shape, it can be concluded that these are caused by air inclusions. This type of macroporosity is quite common in OFCs and has little effect on the material properties [7,21,22].

The three test series with 3D braids show that the average ILSS and flexural strength of 3D braids increase with the Z-fiber content (Table 5). Mean flexural strengths of up to 374 MPa are achieved with the 3D braids (Table 5), with a fiber volume content of up to 27.2%. Elongation at break is in the range of about 0.4% (Figure 17a), thus in the range of that of fabric-reinforced specimens with the same matrix composition [22].

Compared to 2D braids, the ILSS of 3D braids can be increased from 14.8 MPa to up to 24.3 MPa, i.e., by up to 64%, predetermined by the preform structure (Table 6). This increase is mainly attributed to the Z-fiber content; regarding the fiber volume content, a smaller influence is assumed.

Up to this point, two important influences, namely the manufacturing process and the porosity, on the mechanical properties have not been considered. In our previous work [21,22], we showed that the prepreg process (320 ± 24 MPa) lead to higher strengths than the vacuum infiltration process (220 ± 19 MPa). This cannot only be attributed to the higher fiber volume content of the OFCs derived by the prepreg process, which was 42.9% compared to the 38.1% from the vacuum-infiltrated OFCs, but to a more homogeneous infiltration of the fiber bundles during the prepreg process, because the fiber bundles are less compacted during infiltration [21,22]. The interlaminar matrix areas, however, were similar in homogeneity for both processes; therefore, similar ILSS values would be expected, but the ILSS of the OFCs derived from the vacuum infiltration process was not measured. Since 10,000 den fiber bundles are used for the braids, i.e., 2550 single filaments instead of 750 single filaments in the fiber bundles used for the fabric, the homogeneous infiltration of the fiber bundles is even more challenging for OFCs with braided reinforcements. While the damage tolerance of weak-matrix OFCs is based on the high degree of matrix porosity, macropores can have a detrimental effect on mechanical properties, depending on the pore shape as well as its position [21,22]. Since the fiber volume contents of the OFCs discussed in this paper vary over a range from < 20% for 2D-braid-reinforced OFCs to more than 40% for fabric-reinforced OFCs, the comparison of the overall porosity of the OFCs is not useful. Instead, the relative macroporosity, i.e., the calculated content of pores > 1 µm in regard to the overall porosity [22], is used as key figure. The relative macroporosity of the fabric-reinforced OFCs is lowest with 10.8%. This is to be expected, because the prepreg process was developed for fabric reinforcement and the fiber bundle infiltration is easier for fiber bundles with a lower single-filament count. The 2D-braid reinforced sample with an ILSS of 14.8 ± 1.0 MPa has a relative macroporosity of 20.2% and the three 3D-braid-reinforced samples have a mean relative macroporosity of 13. 4 ± 1.3%. While the increase in the macroporosity of the samples with 3D braid reinforcement is most likely due to the higher filament count of the bundles, its distinct increase for the 2D-braid-reinforced sample is due to a combination of a manufacturing process and a filament count which both make homogeneous infiltration of the bundles more difficult. However, since the increased macroporosity predominately appears in the fiber bundles, the effect on the ILSS is deemed less pronounced than the effect of an increase in Z-fiber content.

Guglielmi et al. [41] have shown that the ILSS could also be increased by matrix densification due to annealing, albeit not all samples failed under interlaminar shear afterwards. The ILSS strongly depends on the material system and processing parameters, such as sintering time and temperature, i.e., the comparison of ILSS values is not always straightforward, especially since there can be a trade-off between ILSS and other strength values, such as tensile strength.

## 4. Conclusions

By modifying the 2D and 3D braiding process chain with the aim of processing the fibers as gently as possible, brittle oxide ceramic fibers could also be processed with less damage. For this objective, a bobbin for the 3D braiding process was developed, taking into account existing braiding bobbin concepts. Furthermore, the process control was designed with a focus on fiber-protecting processing, whereby filament breakage could be greatly reduced. In combination with a fiber-protecting process design, filament breakage could be greatly reduced. For the first time, the results showed the possibility of processing highly brittle oxide ceramic fibers on a 3D braider. A Z-fiber content of up to 13.1% in 3D braids results in an interlaminar shear strength (ILSS) which is 64–95% higher than the achievable interlaminar shear strength in two-dimensionally braided or woven reinforced samples with the same Al_2_O_3_-ZrO_2_ matrix composition. The braided samples were infiltrated by a prepreg process and a vacuum-assisted process. The infiltration of the braids is challenging, because the fiber bundles have a high filament count and are compacted due to the braiding process itself, as well as due to the vacuum-assisted process. Since the interlaminar matrix areas are of similar homogeneity for all samples and manufacturing methods, the differences in ILSS between the various types of reinforcement can be mainly attributed to the Z-fiber content.

## Figures and Tables

**Table 1:** Influences on fiber stress and derived solutions.

Fiber-to-Fiber Friction as a Result of a Non-Tangential Pull-Off from the Coil		
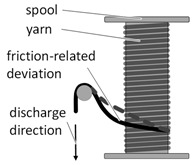 Coulomb formula		Solutions and partial solutions: Baffle. Horizontal bobbin. Slider bobbin with a movable first-deflection roller. Van Reden electric bobbin with a moving sensor-controlled first deflector. Horizontal bobbin with a very high thread eye or ideally without a thread eye at all.
$F_{\mathrm{Rigid}}=\mu \;\cdot {\;F}_{N}$	(2)	
Yarn Damage Due to Friction as a Result of Deflection at Yarn Guiding Elements		
Rolling resistance force		Solutions and partial solutions: Horizontal bobbin with a minimum number of yarn guiding elements. Use of deflection rollers instead of eyelets. Friction reduction through suitable material pairing and geometry. Clean eyelets. Reduction in the wrap angle.
$F_{\mathrm{Roll}}=c_{r}\;\cdot {\;F}_{N}$	(3)	
Euler–Eytelwein formula		
$F_{\mathrm{After}}{=F}_{\mathrm{Before}}\;\cdot \;\left(e^{\mu \gamma }-\;1\right)$	(4)	
Yarn Damage Due to Mass Inertia, Bearing and Gear-Related Frictional Resistance		
Dynamic force balance		Solutions and partial solutions: Low coil mass. No transmission gear. Large radii.
$F_{\mathrm{Fiber}}=\frac{J_{S}}{r}\;{\cdot \ddot{\varphi }}_{S}+c{\left(\varphi \right)}^{\prime }\;\cdot \;\varphi$	(5)	
Filament Ring Formation During Unwinding		
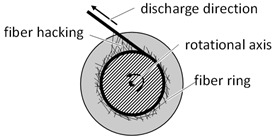		Solutions and partial solutions: Large radii of the coil. Sizing intact. Fiber-friendly winding process.

**Table 2:** Characteristics and the effect of the variables of the factor test space.

Designation		Lower Value (−)	/	Upper Value (+)	Maximum Thread Tension Force	Minimum Thread Tension Force	Settling Length
Pull-off speed	v_A_	0 m/min	→	0.38 m/min	+	+	/
Horn gear speed	n_F_	3.75 min^−1^	→	50 min^−1^	+	+	+
Spring thickness	c_F_	0.12 mm	→	0.15 mm	+	+	+
Yarn eye deflection	l_F_	0 mm	→	38 mm	-	/	/
Rotatability	ω_F_	=0 min^−1^	→	≠0 min^−1^	-	/	/

**Table 3:** Characteristics of the tribological analysis.

Friction Body Material (mat.)	Friction Body Diameter (d_F_)	Fiber Force (F_F_)	Pull-Off Speed (v_A_)
Polished stainless steel (VA)	10 mm	1 N	0.25 m/min
VA with diamond-like carbon (DLC)	30 mm	2 N	1.25 m/min
VA with titanium carbonitride (TiCN)	50 mm	4 N	2.5 m/min
VA with Topocrom Nr. 131 (TOP)	70 mm		

**Table 4:** Influence of spring tension, braiding angle and vibrator setting on the interlaminar shear strength.

Spring Strength (g/ N)	100 g/1 N			200 g/2 N		
Braiding Angle (°)	30°	45°	60°	30°	45°	60°
**Vibrator off, ILSS (MPa)**	14.8 ± 1.0 MPa	13.0 ± 1.1 MPa	11.8 ± 1.0 MPa	13.8 ± 1.1 MPa	12.0 ± 2.2 MPa	10.7 ± 2.7 MPa
**Vibrator on, ILSS (MPa)**	14.1 ± 1.1 MPa	12.1 ± 1.7 MPa	11.1 ± 1.6 MPa	14.8 ± 0.3 MPa	12.1 ± 0.9 MPa	11.1 ± 1.1 MPa

**Table 5:** Interlaminar shear strength and 3-point bending strength of OFCs with 3D braid reinforcement.

Test Series	ILSS (MPa)	Flexural Strength (MPa)	Fiber Volume Content (%)	Z-Fiber Content (%)	Braiding Angle
Sample 1	21.7 ± 4.8	354 ± 48	26.0	7.2	30°
Sample 2	23.7 ± 3.3	358 ± 53	26.1	10.6	30°
Sample 3	24.3 ± 1.7	374 ± 64	27.2	13.1	30°

**Table 6:** Properties of various textile-reinforced OFCs with Nextel^TM^610.

Textile Type	Fiber Angle (°)	Fiber Volume Content (%)	Flexural Strength (MPa)	ILSS (MPa)	Z-Fiber Content (%)
Two-dimensional woven fabric	0/90°	38.1%	220 ± 19 MPa	12.4 ± 2.4 MPa	0%
Two-dimensional braid	30°	30.2%	216 ± 47 MPa	14.8 ± 1.0 MPa	1.5%
Three-dimensional braid	30°	27.2%	374 ± 64 MPa	24.3 ± 1.7 MPa	13.1%
